# Efficacy of Combined Photobiomodulation Therapy with Supplements versus Supplements alone in Restoring Thyroid Gland Homeostasis in Hashimoto Thyroiditis: A Clinical Feasibility Parallel Trial with 6-Months Follow-Up

**DOI:** 10.3390/jpm13081274

**Published:** 2023-08-19

**Authors:** Venera Berisha-Muharremi, Bernard Tahirbegolli, Ruth Phypers, Reem Hanna

**Affiliations:** 1 Faculty of Medicine, University of Prishtina, Bulevardi i Dëshmorëve nn, 10000 Prishtina, Kosovo; venera.berisha@uni-pr.edu; 2 Poliklinika Endomedica, Muharrem Fejza Str. Nr. 84, 10000 Prishtina, Kosovo; 3 Department of Management of Health Institution and Services, Heimerer College, 10000 Prishtina, Kosovo; bernardtahirbegolli@gmail.com; 4 National Sports Medicine Centre, Lagjia e Spitalit nn, 10000 Prishtina, Kosovo; 5 Laser Medicine Centre, 10 Harley Street, London W1G 9PF, UK; ruth@lasermedicine.co.uk; 6 Department of Oral Surgery, King’s College Hospital NHS Foundation Trust, London SE5 9RS, UK; 7 Department of Surgical Sciences and Integrated Diagnostics, University of Genoa, Viale Benedetto XV, 16132 Genoa, Italy; 8 Department of Restorative Dental Sciences, UCL-Eastman Dental Institute, Faculty of Medical Sciences, Rockefeller, University College London, London WC1E 6DE, UK

**Keywords:** autoimmune disease, autoimmune thyroiditis, Hashimoto thyroiditis, immunotherapy, inflammation, oxidative stress, photobiomodulation, ROS, supplements, ultrasound

## Abstract

Hashimoto thyroiditis (HT) is a thyroid-specific autoimmune disorder, triggering hypothyroidism in a population with an adequate dietary intake. Despite the current conventional treatment focuses on the permanent replacement of levothyroxine (LT4) deficiency, it appears that thyroid autoimmunity remains the cause of persistent symptoms in patients with HT, even when they achieve to be euthyroid from a biochemical standpoint. Photobiomodulation (PBM) showed to be an effective therapy in the management of autoimmune diseases, but with limited evidence. Hence, our study was conducted to appraise the efficacy of PBM therapy with supplements in restoring thyroid gland homeostasis in patients with HT compared with supplements alone. Seventy-four female subjects aged between 20 and 50 years old were recruited and divided equally into two groups: PBM and supplements group (group 1); and supplements alone group (group 2). The PBM dosimetry and treatment protocols were as follows: wavelength, 820 nm; power output, 200 mW; continuous emission mode; irradiating time, 20 s per point; fluence, 32 J/cm^2^ per point; treatment frequency, twice a week (excluding weekends); and treatment duration, three consecutive weeks. Whereas, the supplements protocol for both groups was the same, as follows: subjects with a serum level of vitamin D3 <40 ng/dL, who received replacement according to their serum levels, and all the subjects had a daily intake of 100 µg of oral selenium. The biochemical (FT3, FT4, antiTPO and antiTG) and anthropometric measurements were evaluated. Our findings showed significant improvement in group 1 parameters (PBM+ supplements) compared with group 2 (supplements only) in terms of weight loss and reduction in the following parameters: BMI, hip and waist circumference, waist/hip ratio, TSH, antiTPO, antiTG and treatment dose of LT4 (*p* < 0.05). Our results, for the first time, demonstrated an efficacy of PBM delivered at a lower fluence with supplements in restoring thyroid function, anthropometric parameters and lifestyle factors in patients with HT. Hence, extensive studies with a longer follow-up period are warranted.

## 1. Introduction

Hashimoto thyroiditis (HT) is known as chronic lymphocytic thyroiditis (CLT) and chronic autoimmune thyroiditis (CAT). The most common form of thyroid-specific autoimmune disorders is HT, which is characterized by an autoinflammatory state and a lymphocytic infiltration of thyroid follicles [1], triggering hypothyroidism in populations with an adequate dietary intake [2].

### 1.1. Hashimoto Thyroiditis Immunopathogenesis

Cell-mediated autoimmune responses, thyroid peroxidase antibodies (antiTPO) and thyroglobulin antibodies (antiTG) [3] can prompt thyroid follicular cell injury. These can lead to progressive cell destruction, which subsequently results in hypothyroidism [4,5,6]. Despite the fact that the exact etiology of CAT remains unclear, the loss of self-tolerance as a result of a genetic predisposition, in combination with environmental variables such as selenium insufficiency, high iodine intake, smoking and viral infections, has been recognized. The thyroid tissue is destroyed as a result of autoimmune responses, leading to insufficient thyroid gland functionality by which hypothyroidism occurs. HT is commonly associated with immune cell infiltration involving macrophages, antigen-presenting cells, plasma cells and lymphocytes in the thyroid parenchymal tissue [7,8]. Immune responses can lead to the production of thyroglobulin (Tg) and thyroid peroxidase (TPO) auto-antibodies, triggering thyroid cell damage [9,10].

It is noteworthy that microRNAs (miRNAs) are fundamental epigenetic regulators participating in several autoimmune diseases, wherein HT is one of them [11,12]. Hence, a clinical study conducted by Li et al. [11] investigated the pathogenic role of miR-326 and its underlying molecular mechanism in patients with HT. The authors’ findings suggest that the miR-326 effects on the interleukin (IL)-23/IL-23R/Th17 cell axis in patients with HT might be partially due to the targeting of ADAM17. Another clinical study conducted by Li et al. [12] explored the role and the potential mechanism of tissue sEV miRNAs in HT pathogenesis. The results documented the fact that tissue sEV-mediated miR-142-3p transfer can serve as a communication channel between T lymphocytes and thyrocyte cells in HT, favoring HT progression.

### 1.2. Current Treatment Modalities of Hashimoto Thyroiditis

Currently, there is no treatment modality that is based on underlying the pathological mechanism of HT development. Despite the existing conventional treatment focuses on the permanent replacement of the hormone deficiency with levothyroxine (LT4) [13,14], it appears that thyroid autoimmunity remains the cause of persistent symptoms in patients with HT, even though, biochemically, they have achieved to be euthyroid [15].

Several studies showed that most of the subjects who had HT, even in the euthyroid state, experienced body weight excess and metabolic disorders [16,17]. It is noteworthy that obesity is significantly linked to HT and excessive antiTPO. Hence, embracing lifestyle changes with a tailored nutrition plan and supplements are essential in the therapeutic strategy for patients with HT to improve wellbeing and reduce complication rates, which ultimately can enhance their quality of life (QoL). Until now, the standardized diet recommendation to patients with HT is unspecified; nevertheless, gluten-free diet has been advised [18].

Many researches have investigated the link between HT and vitamin D deficiency in recent years [19,20,21,22,23]; therefore, it has been suggested that vitamin D supplementation should be considered [15]. Several studies also showed the beneficial role of prescribing selenium (Se) supplements [24,25], but the evidence remains insufficient [21].

### 1.3. Photobiomodulation-Induced Antioxidant Effect in Restoring Homeostasis of Thyroid Gland

At various stages and in different types of thyroid disorders, a discrepancy between oxidants and antioxidants are observed [26]. The thyroid gland is sensitive to the oxidative process. High levels of reactive oxygen species (ROS) are produced in the thyroid gland in physiological conditions [27]. It was shown that photobiomodulation (PBM) could have a major impact on modulating ROS levels in the thyroid gland [27].

PBM therapy is a non-invasive treatment modality, whereby its mechanism of action is evolving. The light photonic energy is absorbed by the cytochrome C oxidase (photoacceptor) of the mitochondria, generating a cascade of cellular and molecular activities, leading to an upregulation of anti-inflammatory cytokines while modulating ROS and nitric oxide (NO) [28,29,30], promoting analgesic effects [31,32] and regenerating biological tissues [33,34]. Hence, PBM showed to be an effective therapy in the management of autoimmune diseases such as rheumatoid arthritis [35,36].

#### 1.3.1. PBM Improves Thyroid Tissue and Functionality in HT

From molecular and immunomodulation standpoints, PBM can improve thyroid gland functions and reduce antiTPO levels in patients with hypothyroidism caused by CAT [37,38,39]. In healthy animals, PBM therapy can improve thyroid microcirculation [40,41] and elevate the serum concentrations of T3 and T4 [42]. These positive outcomes coincided with several clinical studies, indicating that thymus and thyroid (vasculature) irradiated with PBM can induce systematic immunomodulatory effect [43] to improve blood microcirculation and modulate T3 and T4 hormones in patients with CAT [44,46].

It was also shown that PBM therapy can accelerate thyroid recovery and functionality in animal models induced with thyroid damage 14 days after ionizing radiation. The activity of the super-oxide dismutase enzyme downregulated significantly during the first week of PBM therapy. This reduction enabled the thyroid tissue to detoxify the free radicals and recover its function by decreasing oxidative stress (OS) [47].

Moreover, the findings of another *in vivo* animal study showed the effects of laser PBM on the shape and structure of thyrocytes and thyroid follicles, as well on the thyroid size and volume of irradiated male rats induced with hypothyroidism. This indicates an improvement in the secretion of the thyroid hormones and an increase in the vascularization [48], resulting in the regeneration of the thyroid gland follicular cells [39,49].

#### 1.3.2. PBM Immunomodulates Inflammatory Cytokines Induced by HT

As mentioned before, inflammatory cytokines such as; tumor necrosis factor alpha (TNF-α) and interferon gamma (IFN-γ) play a crucial role in AIT pathogenesis. Hence, an increase in the levels of anti-inflammatory cytokines such as transforming growth factor β (TGF-β) modulates the inflammatory microenvironment and plays a major role in self-tolerance maintenance [50]. An increase in the serum concentration of TGF-β could inhibit autoimmune diseases, including CAT [51]. It was also shown that PBM can stimulate TGF-β production in AIT along with a reduction in the levels of pathogenic antiTPO [38].

### 1.4. Rationale in Conducting This Study

Although PBM is safe with no risk of developing malignant nodules [52] and can improve thyroid functions and increase thyroid hormones [38,53,54,55], PBM dosimetry (power and irradiation time) and treatment protocol need to be carefully chosen to achieve optimal outcomes [56]. Hence, the present study is aimed to evaluate the efficacy of near infra-red (NIR)-PBM with supplements versus supplements alone in the management of patients with HT. The study’s objectives are as follows: (1) to establish optimal laser PBM treatment protocol; (2) to increase thyroid hormones; and (3) to improve patients’ lifestyle factors.

## 2. Materials and Methods

### 2.1. Study Design

An experimental open label non-randomized interventional clinical trial was conducted to evaluate PBM efficacy with supplements in restoring thyroid gland homeostasis in patients with HT compared with supplements alone. The study was performed at Poliklinika Endomedica, Prishtina, Kosovo where the subjects were recruited in the period between March 2022 and March 2023. The study’s blinding strategies were as follows: blinding of outcome adjudicators and data collectors. The performer is a clinician with a wealth of experience in the fields of laser therapy and endocrinology. The study was conducted in accordance with the Declaration of Helsinki and the study protocol was approved by the Ethics Committee of the Institutional Review and Ethics Committee of Faculty of Medicine, University of Prishtina, Kosovo (project identification code: 2687).

#### 2.1.1. Population (P), Intervention (I), Comparison (C) and Outcome (O)—PICO

P: Female adults aged between 20 and 50 years old who were diagnosed with HT based on clinical and diagnostic criteria [57,58,59].

I: λ820 nm laser PBM and supplements.

C: Supplements with no PBM therapy.

O: Outcomes evaluation with biochemical and anthropometric measurements.

#### 2.1.2. Eligibility Criteria

##### Inclusion Criteria

Adult female subjects aged between 20 50 yearold who were diagnosed with HT according to the following specific criteria: (1) high serum levels of thyroid autoantibodies antiTPO and/or antiTG (antiTPO ref. range <34 IU/mL; antiTG ref range <115 IU/mL) and (2) ultrasound findings of HT (GE Logiq V5 Ultrasound) [57,58,59].

##### Exclusion Criteria

- Patients with any known autoimmune diseases except HT or any other treatment except LT4.
- Adult female aged <20 and >50 years old.
- Adult male of any age group.
- Patients previously treated with radioiodine.
- Patients taking immunosuppressants, immunostimulants and any drug that could interfere with the production, transport and metabolism of thyroid hormones (e.g., corticosteroids, lithium, amiodarone).
- Subjects with thyroid nodules or ectopic thyroid or thyroid hypoplasia.
- Hypothyroidism stemming from postpartum thyroiditis (up to 18 months after gestation).
- A history of Graves’ disease.
- Tracheal stenosis.
- Pregnant women.
- Subjects with a history of exposure to ionizing irradiation and/or neoplasia in the cervical region.
- Patients with previous thyroid surgery.
- Patients with a serious illness (e.g., kidney and liver failure, cancer, stroke).

#### 2.1.3. Patient Cohort

**Figure 1:**
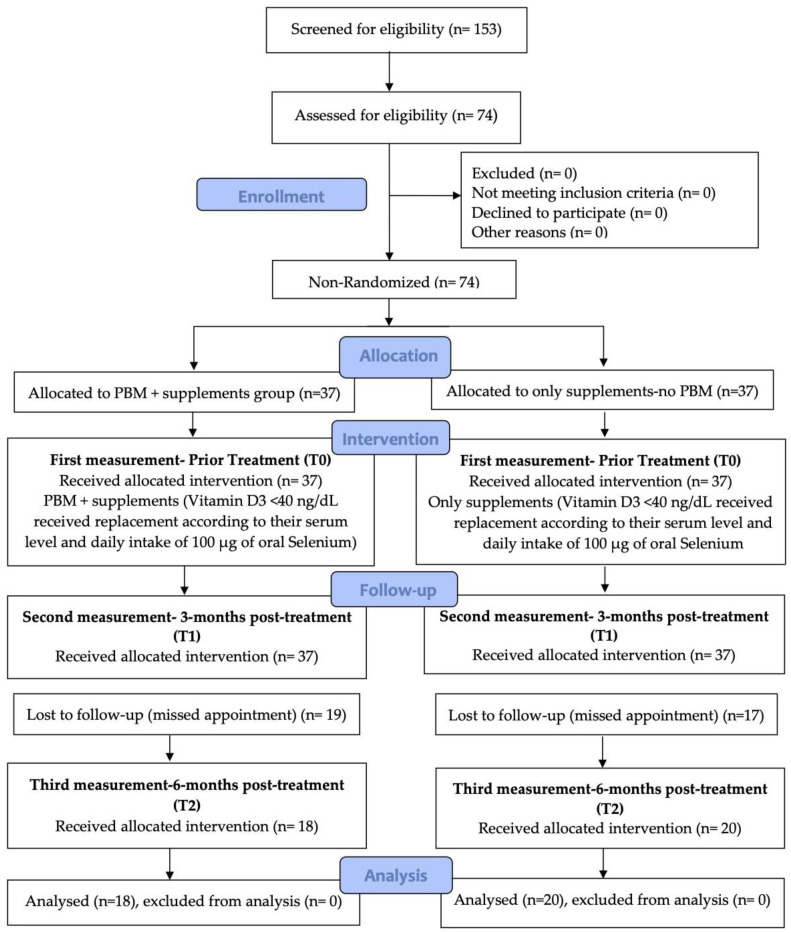
Flow chart showing the experimental design of the study (at T0, there were 37 recruited subjects in group 1 and group 2, but at six months after treatment (T2), the remaining subjects were 18 and 20, respectively).

After taking the eligibility criteria into consideration, 74 female subjects were recruited and divided into two groups: group 1 received PBM with supplements (*n* = 37) and group 2 received only supplements (*n* = 37) (Figure 1). We chose only female subjects between 20 and 50 years old in our study for the following reasons: (1) to eliminate the influencing factors and achieving homogeneous unbiased results; (2) to define population along with significance level and power statistically (sample size), as the evidence-based science and practice documented that female patients with HT are more predominant than males at a ratio of 7–10:1, respectively, due to genetic susceptibility, X chromosome inactivation patterns modulated by environmental factors, as well microbiome composition, leading to an imbalance in self-tolerance mechanisms [3,8]. The study group assignments and outcome evaluation strategy depended on the individual patient preferences rather than on randomization.

The supplements protocol for both groups was similar, as follows: patients with a serum level of vitamin D3 <40 ng/dL received replacement according to their serum levels, and all the subjects had a daily intake of 100 µg of oral selenium. An appropriate dose of LT4 and vitamin D3 replacement for each patient was determined before their enrolment in the study by an endocrinologist who was not involved in the study. Each subject was given a patient advice leaflet, asking to avoid food with gluten and sugar ingredients.

All the recruited subjects signed an informed written consent after the proposed treatments were explained.

#### 2.1.4. Treatment Protocols

##### Ultrasound

Ultrasound (GE Logiq V5, GE Healthcare, secured from Solingen, Germany) was employed to define the anatomical borders of the thyroid gland on the skin where eight target points (four points on each lobe of thyroid gland) were marked with a surgical pen with a distance of 1 cm apart from each other.

##### PBM Protocol

Table 1 shows laser device specifications, the study’s laser parameters and the treatment protocols. A single laser probe (Omega XP, Laser Systems Limited, Essex, UK) delivering a photonic energy of 820 nm at a therapeutic power output of 200 mW (measured with PM160T-power meter, Newton, NJ, USA) in a continuous emission mode was employed. The irradiation time per point was 20 s. The total number of irradiation points was eight. The laser probe was held in contact and at 90° in relation to the target tissue, delivering a fluence of 32 J/cm^2^ per point, where the total fluence of 256 J/cm^2^/session (160 s/session) was delivered over the thyroid. The treatment protocol was a total of six sessions based on a frequency of twice a week (excluding weekends) for three consecutive weeks.

### 2.2. Outcomes Measures

#### 2.2.1. Primary Outcomes Measurement

The primary outcomes were to improve thyroid gland functions after PBM in terms of a reduction in TSH level, an increase in FT4 level and a reduction in LT4 dose required for substitution in group 1 (PBM with supplements) compared with group 2 (supplements alone—no PBM).

#### 2.2.2. Secondary Outcomes Measurement

The secondary outcomes were to evaluate the weight managements in terms of body mass index (BMI) reduction and waist and hip circumferences in both groups.

### 2.3. Assessment Tools Utilized to Evaluate Outcome Variables

#### 2.3.1. Biochemical Measurement

The serum levels of TSH, FT4, FT3, antiTPO and antiTG (ElectroChemiLuminescence technology for immunoassay, Cobas e 411 Roche-Hitachi nalyzer, Hitachi High-Technologies Corporation 1-24-14 Nishi-Shinbashi, Minato-ku, Tokyo 105-8717 Japan) [60] were evaluated prior to the treatment (T0), at three- months (T1) and six-months (T2) after the treatment.

#### 2.3.2. Lifestyle Factors

Weight (kg), height (m), waist circumference (cm), hip circumference (cm), calculated BMI (weight (kg) per height (m^2^)) and waist/hip ratio measurements were all evaluated prior to the treatment (T0) at three- months (T1) and six-months (T2) after the treatment.

### 2.4. Statistical Analysis

The variables were calculated with the G-power program in post hoc, in the ANOVA axis for three repeated measurements. It was estimated that the research sample with 18 participants in one group and 20 participants in the second group with an effect size f of 0.25 and 0.05 α, respectively, had a power of 0.92 1-β. The IBM SPSS v21.0 package program was used to examine the data. Continuous variables were described using the mean and standard deviation (SD) or the median and interquartile range (IQR), whereas categorical variables were summarized using the frequency (n) and percentage (%). Independent groups t-test or Mann–Whitney U test, paired samples t-test or Wilcoxon signed rank test and GLM Repeated Measurements or Friedman Repeated Measures Analysis were utilized to evaluate the differences across the variables. The *p* value of <0.05 was considered statistically significant.

## 3. Results

The quantitative analysis of our data showed that there was no statistically significant difference in age, heigh, weight, BMI, waist circumference, hip circumference, TSH, FT4, antiTG and the dose of LT4 used for treatment in the first visit at T0 (*p* > 0.05) between both groups (Table 2). The mean age of the subjects was 38.5 ± 5.3 years old.

It is noteworthy that at T0, there were 37 subjects in each group, but at the third measurement, six months post-treatment (T2), the remaining subjects were 38, with 18 in group 1 and 20 subjects in group 2 due to missed appointments (Figure 1).

The analysis for the repeated measures showed a statistically significant improvement in losing weight, decreasing BMI, hip and waist circumference, waist/hip ratio, TSH, antiTPO, antiTG and a decrease in the treatment dose of LT4 (*p* < 0.05) among patients in group 1 compared to group 2 (Table 3), where there were no statistically significant improvements observed in weight loss, BMI, waist and hip circumference, waist/hip ratio, TSH, antiTPO and antiTG (*p* > 0.05).

**Figure 2:**
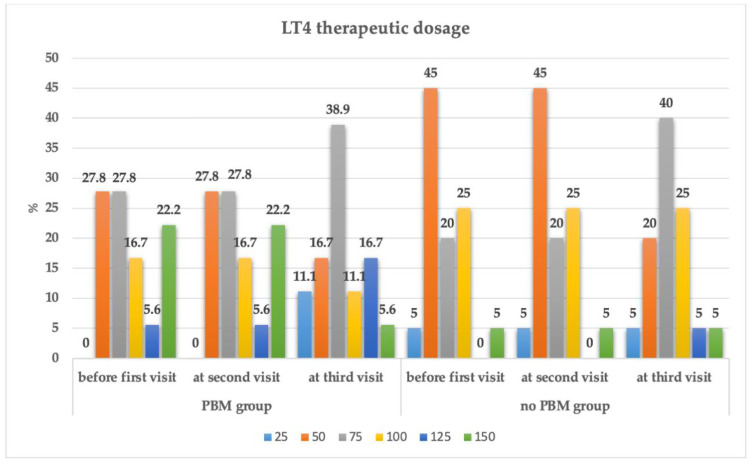
The changes in the LT4 treatment dosage in both groups at first visit (T0) pre-treatment; second visit (T1) three-months post-treatment; third visit (T2) six-months post-treatment. The numbering on the X-axis of the graph represents the interventional groups and their associated timepoints, as follows: 1—PBM group at the first visit; 2—PBM group after the second visit; 3—PBM group after the third visit; 4—no-PBM group at the first visit; 5—no-PBM group after the second visit and 6—PBM group after the third visit. The Y-axis represents the percentage of the changes in LT4 therapeutic dosage. The figures shown on the top of each column represents the percentage of the changes in LT4 at different timepoints for each group.

**Figure 3:**
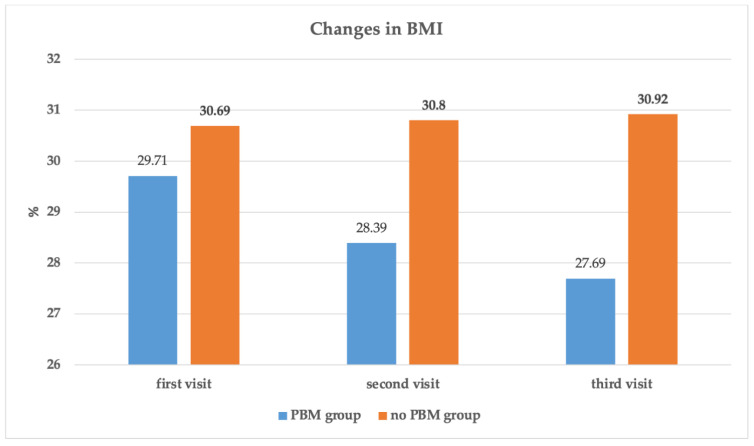
The changes in the BMI in both groups at first visit (T0) pre-treatment; second visit (T1) three-months post-treatment; third visit (T2) six-months post-treatment. The figures shown on the top of each column represents the percentage of the changes in BMI at different timepoints for each group.

Figure 2 shows the changes in the percentage of patients’ LT4 therapy dosage among T0, T1 and T2 visits in group 1 compared with group 2. There was a statistically significant reduction in the T4 needs in group 1, whereas Figure 3 presents the changes in the BMI measurements at T0, T1 and T2 in group 1 compared with group 2. A significant reduction in the BMI in group 1 was observed.

The analysis between groups that was produced for the time*group factor showed a statistically significant difference in weight, BMI, hip and waist circumference, TSH, FT4, FT3, antiTPO, antiTG and in the treatment dose of LT4 (*p* < 0.05) (Table 4). In group 1, the number of female subjects that needed 150 µg of LT4 decreased from four to one dose (Table 5).

## 4. Discussion

Our results demonstrated PBM to be positive and statistically significant results in improving thyroid gland function in patients with HT in terms of a reduction in the TSH level, an increase in FT4 level and a reduction in the levels of antiTPO and antiTG antibodies. Despite our findings documented the need for LT4 replacement was reduced, our study importantly showed, for the first time, a statistically significant improvement in overweight management by reducing the BMI and waist and hip circumferences in the PBM group compared to the non-PBM group, whereby no statistically significant improvement was observed in the anthropometric measurements of TSH, antiTPO and antiTG. In contrast, a clinical study conducted by Ercetin et al. showed a significant reduction in antiTPO levels in both groups (only PBM (group 1) and only supplements (group 2) groups), but the antibody levels in group 1 were significantly lower than group 2.

All the abovementioned results are indicative of PBM effects related to its anti-inflammatory contribution. Various *in vivo* animal studies utilizing rats as animal models showed that laser PBM inhibits proinflammatory cytokine production such as TNF-α, IL-1 β, IL-2, IL-6, IL-8 and IFN-c by inhibiting the gene expression [61,62]. It is well-documented that there is a relationship between IFN-c and TNF-a, producing type 1 T helper cells and high levels of antiTPO; hence, PBM effects on those proinflammatory cytokines can justify the reduction in the levels of the antibody [63]. Additionally, PBM immunomodulatory and inflammatory effects have proven to downregulate the inflammatory cytokines, to regulate the release of both ROS and NO and to promote the synthesis of antioxidant molecules and growth factors, assisting in damage tissue repair and regeneration [64].

In terms of PBM dosimetry and treatment protocols, our study employed a novel protocol with a low fluence, which is in agreement with a study conducted by Ercetin et al. [55] who utilized a fluence of 28.57 J/cm^2^. In contrast, three studies that were conducted by the same Brazilian research group employed PBM therapy with a high fluence that ranged between 37 and 707 J/cm^2^ in the management of patients with CAT, but showed contradictory results [38,39,58]. In their first preliminary study of 15 subjects conducted by Höfling et al. [39], the authors utilized the following PBM dosimetry and treatment protocol: 830 nm, 50 mW, CW, twice a week, 10 sessions (whereby the irradiation technique was either punctual technique (eight patients) or sweep technique (seven patients)) and with fluence ranging between 38 and 108 J/cm^2^. The results indicated that PBM can prompt improvement in the thyroid function and the patients experienced a decreased need for LT4, as well as a reduction in their levels of antiTPO. Their second study was a randomized placebo-controlled trial of 43 patients with CAT with a 9-month follow-up conducted by Höfling et al. [38], utilizing the same PBM dosimetry of the previous study, but with an irradiation time of 40 s and a higher fluence of 707 J/cm^2^. The findings showed that the antiTPO concentrations were reduced in both groups (PBM and control groups) and concluded that PBM was effective in reducing TPOAb-mediated autoimmunity. On the other hand, their third study, conducted by Höfling et al. [57], utilized the same dosimetry and the fluence of the second study, but no significant difference was observed, indicating that post-PBM, antiTPO reduction has a limited effect over time, and hence, further studies with large data were suggested.

It is noteworthy that the evidence-based science and practice highlighted that PBM with a high fluence leads to inhibitory effects rather than biostimulatory effects [64]. This is indicative that our PBM dosimetry with low fluence is valid and justified.

Moreover, our study validated the PBM efficacy with supplements in improving thyroid function, which is in agreement with previous studies [49,55]; however, none of those studies addressed the impact of utilizing PBM of low fluence on anthropometric values (BMI, waist and hip circumferences), which have a great impact on improving HT symptoms and patients’ lifestyle factors. Ultimately, our finding, for the first time, showed an improvement in the management of overweight with a reduction in BMI, waist circumference and hip circumference in the group treated with PBM compared to the non-PBM group, which is a novelty and very fundamental, since patients who received only supplements even though they became euthyroid continued to have symptoms, among which weight management was very difficult.

PBM therapy evidenced to be a non-invasive and safe interventional tool in increasing thyroid hormone levels and improving thyroid function [36,46,53]. Additionally, it is noteworthy that no adverse effects were reported in our study, which is in agreement with the well-documented literature that PBM has no risk in developing malignant nodes even after a long-term follow-up of 6 years after PBM therapy [57].

Despite the limitations of our study in terms of its moderate sample size, our results validated the effectiveness of combining PBM with supplements compared with administering supplements alone, suggesting the PBM synergetic effects in restoring the thyroid gland homeostasis in HT based on six months follow-up. Moreover, our positive results validated our PBM dosimetry and treatment protocol, as well as our robust assessment tools, including anthropometric measurements, which ultimately are reproducible.

## 5. Conclusions and Future Direction

Our results, for the first time, showed that PBM therapy is effective not only in improving thyroid gland function, but also in reducing the level of antibodies that are responsible for damaging thyroid gland structure in patients with HT, as well as reducing the need for LT4 replacement and most importantly in reducing excessive weight that persists in patients with HT even in the euthyroid state.

Our encouraging results demonstrated the efficacy of PBM when it is delivered at a lower fluence in conjunction with supplements in the treatment of HT. Hence, extensive studies with longer follow-up periods are warranted.

## Figures and Tables

**Table 1:** The laser device specifications, PBM laser parameters and treatment protocols.

Manufacturer	Omega XP
Semiconductor materials (emitter type)	GaAIAs
Probe design	Single probe
Beam delivery system	3B laser
Laser-aiming beam	None
Wavelength	820 nm
Operating emission mode	Continuous wave (CW)
Polarization	Linear
Therapeutic power output	200 mW
Fluence (dose)	32 J/cm^2^ per point
Irradiation time per point	20 s per point
Total number of irradiated points around thyroid gland	8 points
Total of fluence per session	256 J/cm^2^ per session
Total irradiation time per session	160 s
Time interval	Relatively two days, excluding weekends
Treatment frequency	Twice a week
Total treatment sessions	Six sessions
Treatment duration	Three consecutive weeks
Scanning technique	Stationary application
Light-tissue distance	In contact with the skin

**Table 2:** The baseline comparison of some anthropometric and laboratory parameters between group 1 (PBM therapy and supplements) and group 2 (only supplements with no PBM therapy) before the treatment (T0). No significant differences between the two groups at T0 (first visit before the treatment) were observed.

Variables	Group 1 (*n* = 18) Mean ± SD or Median (IQR)	Group 2 (*n* = 20) Mean ± SD or Median (IQR)	*p* Value
Age (yrs)	39.78 ± 4.40	37.30 ± 5.91	t = 1.452, *p* = 0.155
Height (cm)	1.64 ± 0.05	1.65 ± 0.05	t = 0.588, *p* = 0.560
Weight (kg)	80.00 ± 9.63	83.57 ± 9.61	t = 1.142, *p* = 0.261
BMI (kg/m^2^)	29.71 ± 3.36	30.69 ± 3.13	t = 0.991, *p* = 0.357
Waist (cm)	100.39 ± 9.54	105.45 ± 9.84	t = 0.999, *p* = 0.117
Hip (cm)	112.22 ± 7.82	116.55 ± 6.71	t = 0.780, *p* = 0.075
Waist/Hip	0.90 ± 0.06	0.90 ± 0.06	t = 0.681, *p* = 1.000
TSH	3.50(1.94–4.65)	2.92 (1.38–3.80)	U = 134.00, *p* = 0.186
FT4	1.34 ± 0.27	1.53 ± 0.37	t = 1.722, *p* = 0.094
FT3	3.70 (2.48–4.21)	4.10 (3.55–5.08)	U = 247.50, *p* = 0.048
antiTPO	523.60 (291.75–652.55)	219.75 (70.45–538.97)	U = 111.50, *p* = 0.044
antiTG	58.00 (9.05–349.75)	39.28 (18.04–198.25)	U = 166.00, *p* = 0.916
Dose of LT4 prior to treatment (T0)	75.00 (50.00–131.25)	62.50 (50.00–100.00)	U = 126.00, *p* = 0.119

**Table 3:** Comparison among the follow-up anthropometric and the laboratory parameters within the groups. The letters above *p* value represent the following: ^a^, comparison between first and second measurements; ^b^, comparison between second and third measurements; ^c^, comparison between the first and the third measurements.

Interventional Groups	Variables	Measurements			Statistical Analysis Significant/Statistically Insignificance			
		First Mean ± SD or Median (IQR)	Second Mean ± SD or Median (IQR)	Third Mean ± SD or Median (IQR)		*p* ^a^	*p* ^b^	*p* ^c^
**Group 1**	Weight (kg)	80.00 ± 9.63	76.41 ± 9.16	74.59 ± 8.57	F = 51.479, *p* < 0.0001	<0.0001	<0.0001	<0.0001
	BMI (kg/m^2^)	29.71 ± 3.36	28.39 ± 3.24	27.69 ± 2.96	F = 48.097, *p* < 0.0001	<0.0001	<0.0001	<0.0001
	Waist (cm)	100.39 ± 9.54	94.50 ± 9.79	93.44 ± 9.44	F = 24.262, *p* < 0.0001	<0.0001	0.030	<0.0001
	Hip (cm)	112.22 ± 7.82	109.61 ± 7.00	108.89 ± 7.14	F = 18.953, *p* < 0.0001	0.001	0.061	<0.0001
	Waist/Hip	0.90 ± 0.06	0.87 ± 0.07	0.87 ± 0.07	F = 5.667, *p* = 0.029	0.029	NA	0.029
	TSH	3.50 (1.94–4.65)	0.85 (0.10–1.27)	1.25 (0.46–1.51)	x^2^ = 24.602, *p* < 0.0001	<0.0001	0.147	0.001
	FT4	1.34 ± 0.27	3.08 ± 1.74	1.85 ± 0.62	F = 11.908, *p* = 0.001	<0.0001	0.007	0.011
	FT3	3.70 (2.48–4.21)	5.8 (5.35–6.95)	5.15 (4.77–5.20)	x^2^ = 27.070, *p* < 0.0001	<0.0001	0.001	0.001
	antiTPO	523.60 (291.75–652.55)	100.15 (78.00–127.95)	88.25 (62.25–129.50)	x^2^ = 23.111, *p* < 0.0001	<0.0001	0.102	<0.0001
	antiTG	58.00 (9.05–349.75)	36.60 (20.00–143.77)	44.15 (22.85–89.47)	x^2^ = 5.765, *p* = 0.056	0.025	0.433	0.044
	Dose of LT4	75.00 (50.00–131.25)	75.00 (50.00–131.25)	75.00 (50.00–106.25)	x^2^ = 16.000, *p* < 0.0001	1.000	0.005	0.005
**Group 2**	Weight (kg)	83.57 ± 9.61	83.86 ± 9.94	84.17 ± 9.99	F = 2.502, *p* = 0.095	0.293	0.084	0.092
	BMI (kg/m^2^)	30.69 ± 3.13	30.80 ± 3.25	30.92 ± 3.23	F = 2.801, *p* = 0.073	0.286	0.059	0.074
	Waist (cm)	105.45 ± 9.84	105.70 ± 9.96	105.95 ± 10.15	F = 1.727, *p* = 0.204	0.398	0.056	0.163
	Hip (cm)	116.55 ± 6.71	116.85 ± 6.81	116.90 ± 6.83	F = 2.424, *p* = 0.133	0.163	0.330	0.110
	Waist/Hip	0.90 ± 0.06	0.90 ± 0.06	0.90 ± 0.06	F = 0.000, *p* = 1.000	1.000	NA	1.000
	TSH	2.92 (1.38–3.80)	3.35 (2.45–4.25)	3.05 (2.50–4.05)	x^2^ = 4.785, *p* = 0.091	0.025	0.926	0.059
	FT4	1.53 ± 0.37	1.36 ± 0.38	1.28 ± 0.20	F = 3.328, *p* = 0.053	0.018	0.519	0.024
	FT3	4.10(3.55–5.08)	3.90 (3.1–4.44)	3.80 (2.84–4.25)	x^2^ = 7.620, *p* = 0.022	0.012	0.687	0.028
	antiTPO	219.75 (70.45–538.97)	167.50 (92.35–360.75)	171.85 (87.12–443.60)	x^2^ = 0.700, *p* = 0.705	0.263	1.000	0.478
	antiTG	39.28 (18.04–198.25)	44.10 (17.92–276.62)	37.55 (17.95–341.75)	x^2^ = 1.200, *p* = 0.549	0.467	0.970	0.737
	Dose of LT4	62.50 (50.00–100.00)	62.50 (50.00–100.00)	75.00 (56.25–100.00)	x^2^ = 14.000, *p* = 0.001	1.000	0.005	0.008

**Table 4:** The comparison of some anthropometric and laboratory parameters between group 1 (PBM+ supplement) and group 2 (supplement, no PBM) at T0 (prior to treatment), T1 (second measurement, 3 months post-treatment) and T2 (third measurement, 6-months post-treatment).

Variables	Time*Group Factor	
	F	*p* Value
Weight (kg)	54.024	<0.0001
BMI (kg/m^2^)	52.073	<0.0001
Waist (cm)	28.310	<0.0001
Hip (cm)	23.284	<0.0001
Waist/Hip	1.038	0.315
TSH	22.829	<0.0001
FT4	14.023	<0.0001
FT3	30.290	<0.0001
antiTPO	19.083	<0.0001
antiTG	4.915	0.028
Dose of LT4	23.932	<0.0001

**Table 5:** The changes in the LT4 treatment dosage between the second visit (T1) where the data are presented in rows and the third (T2) visit where the data are presented in columns.

**LT4 at second visit (T1)**	**LT4 at Third Visit (T2)**								
	**Interventional Group**	Dose	25	50	75	100	125	150	Total
	**Group 1** **PBM + supplements**	50	2(11.1)	3(16.3)	0(0.0)	0(0.0)	0(0.0)	0(0.0)	5(27.8)
		75	0(0.0)	0(0.0)	5(27.8)	0(0.0)	0(0.0)	0(0.0)	5(27.8)
		100	0(0.0)	0(0.0)	2(11.1)	1(5.6)	0(0.0)	0(0.0)	3(16.7)
		125	0(0.0)	0(0.0)	0(0.0)	1(5.6)	0(0.0)	0(0.0)	1(5.6)
		150	0(0.0)	0(0.0)	0(0.0)	0(0.0)	3(16.7)	1(5.6)	4(22.2)
	**Total**		2(11.1)	3(16.7)	7(38.9)	2(11.1)	3(16.7)	1(5.6)	18(100.0)
	**Group 2** **Only supplements,** **no PBM**	25	1(5.0)	0(0.0)	0(0.0)	0(0.0)	0(0.0)	0(0.0)	1(5.0)
		50	0(0.0)	4(20.0)	5(25.0)	0(0.0)	0(0.0)	0(0.0)	9(45.0)
		75	0(0.0)	0(0.0)	3(15.0)	1(5.0)	0(0.0)	0(0.0)	4(20.0)
		100	0(0.0)	0(0.0)	0(0.0)	4(20.0)	1(5.0)	0(0.0)	5(25.0)
		150	0(0.0)	0(0.0)	0(0.0)	0(0.0)	0(0.0)	1(5.0)	1(5.0)
	**Total**		1(5.0)	4(20.0)	8(40.0)	5(25.0)	1(5.0)	1(5.0)	20(100.0)

## Data Availability

All the data are available in the text.
